# Psychiatric symptoms and pregnancy distress in subsequent pregnancy after spontaneous abortion history

**DOI:** 10.12669/pjms.325.10909

**Published:** 2016

**Authors:** Elahe Haghparast, Mahbobeh Faramarzi, Ramezan Hassanzadeh

**Affiliations:** 1 Elahe Haghparast, Department of Psychology, Islamic Azad University, Sari, Iran; 2 Mahbobeh Faramarzi, Infertility and Reproductive Health Research Center, Babol University of Medical Sciences, Babol, Iran; 3 Ramezan Hassanzadeh, Department of Psychology, Islamic Azad University, Sari, Iran

**Keywords:** Abortion, Psychiatric symptoms, Pregnancy, Distress

## Abstract

**Objectives:**

Spontaneous abortion is one of the most important complications of pregnancy with short and long adverse psychological effects on women. This study assesses the implications of a spontaneous abortion history has on women’s psychiatric symptoms and pregnancy distress in subsequent pregnancy less than one years after spontaneous abortion.

**Methods:**

A case-control study was conducted on pregnant women of Babol city from September 2014 to May 2015. In this study, 100 pregnant women with spontaneous abortion history during a year ago and 100 pregnant women without spontaneous abortion history were enrolled. All the participants in two groups completed the Symptom Checklist-90-Revised (SCL-90-R), and pregnancy Distress Questionnaire (PDQ).

**Results:**

Women with spontaneous abortion history had significantly higher mean of many subscales of SCL-90 (depression, anxiety, somatization, obsessive-compulsiveness, interpersonal sensitivity, psychoticism, hostility, paranoid, and Global Severity Index) more than women without spontaneous abortion history. Also, women with spontaneous abortion history had significantly higher mean of two subscales of PDQ concerns about birth and the baby, concerns about emotions and relationships) and total PDQ more than women without spontaneous abortion history.

**Conclusion:**

Pregnant women with less than a year after spontaneous abortion history are at risk of psychiatric symptoms and pregnancy distress more than controls. This study supports those implications for planning the post spontaneous abortion psychological care for women, especially women who wanted to be pregnant during 12 month after spontaneous abortion.

## INTRODUCTION

Spontaneous abortion is the unexpected loss of pregnancy before the 20th week. It occurs in 12-30% of all confirmed pregnancies.^1^ Pregnancy period is accompanied with high level of emotional stress^2^ and spontaneous abortion is both physically and psychologically a traumatic experience.^3^ A longitudinal study reported a significant proportion of patient’s psychological morbidity shortly after spontaneous abortion.^4^ The majority of women experience grief, depression or both after spontaneous abortion.^5^ Some women also experience anxiety, anger, posttraumatic stress, and guilt about future childbearing.^6^ High levels of anxiety, depression, and grief may persist in some women long after physical recovery has occurred.^7^

The symptoms of anxiety and depression, which occur after spontaneous abortion, may also extend into a subsequent pregnancy.^8^ As the majority of women will become pregnant again within 18 months^9^, the effect of spontaneous abortion on subsequent pregnancy is a great concern. Women expressed the need for more supportive care from professionals not only after spontaneous abortions but also in a new pregnancy.^10^

Although during the last decade there has been a fast growing literature on the emotional impact of spontaneous abortion elevated rates of psychological morbidity in the early months following the pregnancy loss, few studies assessed the adverse effects of prior spontaneous abortion on increasing levels of psychiatric symptoms on subsequent pregnancy. A study reported that women with a spontaneous abortion history had significantly higher pregnancy-specific anxiety at trimester one than women with no spontaneous abortion history.^11^

As adverse effects of prior spontaneous abortion on increasing levels of psychiatric symptoms on subsequent pregnancy less than 12 months is controversial in previous studies,^4^,^8^ the aim of this research was to assess the psychiatric symptoms and pregnancy distress in subsequent pregnancy less than one years after spontaneous abortion history. We compared the psychiatric symptoms and pregnancy distress in pregnant women with and without spontaneous abortion history.

## METHODS

This cross-sectional (case-control) study was conducted between September 2014 to May 2015 in pregnant women of seven private and public health prenatal clinics of Babol city. Available sampling was utilized to recruit the pregnant women based on their eligibility.

Eligible women who were more than 18 years old, with more than five years of education, and willingn to participate in the study were invited to enter the study. Socio-demographic variables and pregnancy information were obtained during the interview. Subjects were considered the spontaneous abortion history group (case) via self-reported history of spontaneous abortion during a prior pregnancy which terminated less than a year ago. One hundred patients diagnosed with spontaneous abortion history and uncomplicated pregnancy agreed to participate in the study. A control group, consisting of 100 patients with no history of spontaneous abortion, no uncomplicated pregnancy was selected from the patients admitted to other outpatient clinics during the study period. Two hundred pregnant women participated in the study.

The researcher distributed the questionnaires to the respondents during prenatal visit. All the participants in two groups completed the Symptom Checklist-90-Revised (SCL-90-R), and pregnancy Distress Questionnaire (PDQ). Ethical approval was granted by the Medical Education Ethic of Committee at Islamic Azad University.

## Scales and Measurement

### SCL-90-R

The psychological symptoms were assessed with the widely-used Symptom Checklist-90-Revised (SCL-90-R), a self-rating inventory with 9 clinical scales for somatization, interpersonal sensitiveness, obsessive-compulsiveness, hostility, phobic anxiety, paranoid ideation, depression, anxiety and psychoticism. Global Severity Index (GSI) were considered to be the measures of overall psychological symptoms.

### Pregnancy Distress Questionnaire

The PDQ (designed by Yali and Lobel, 1999) is a reliable and valid measure of pregnancy-specific stress in assessing pregnancy-specific stress (maternal fears and worries related to pregnancy). The PDQ is a 12-item scale that provides three subscales: concerns about birth and the baby, concerns about weight/body image, and concerns about emotions and relationships. Each item is rated on a 5-point Likert scale ranging from 0 (not at all) to 4 (extremely). PDQ has a possible range of 0–48 for a total score, 0–24 for birth concerns, and 0–12 for both body concerns and relationship concerns.^12^

### Statistical Analysis

Two groups of subjects were compared in demographic status using Chi square test. Two Multivariate Analysis of Variance Models (MONOVAs) was used to compare the mean of all variables between two groups.

## RESULTS

There was no difference between pregnant women with miscarriage history and without miscarriage history in characteristic baseline. Table-I. The mean of the variables are shown in Table-II. MANOVAs on SCL-90-R subscales (Table-III) revealed a significant effect for the some psychiatrict symptoms. Women with miscarriage history had significantly higher mean of many subscales of SCL-90-R (depression, anxiety, somatization, obsessive-compulsion, interpersonal sensitivity, psychoticism, paranoid, hostility, and Global Severity Index) more than women with no history of miscarriage. There were no differences between case and control patients in term of phobia. MANOVAs on PDQ subscales revealed a significant effect for the two subscales and total PDQ. Women with miscarriage history had significantly higher mean of two subscales of PDQ concerns about birth and the baby, concerns about emotions and relationships and total PDQ more than women without miscarriage history. There were no differences between case and control patients in term of concerns about weight/body image.

**Table-I:** Demographic Factors of the Population.

*Variables*	*Miscarriage history*			*P-Value*

	*Yes*	*No*	*Total*
*Age (Years)*				
17-20	13 (13)	13 (13)	26 (13)	0.81
21-30	58 (58)	65 (65)	123 (61.5)	
≥ 31	29 (29)	22 (22)	51 (25.5)	
*Job*				
Employee	44 (44)	35 (35)	79 (39.5)	0.19
Non employee	56 (56)	65 (65)	121 (60.5)	
*Education (Years)*				
≤ 12	46 (46)	52 (52)	98 (49)	0.62
> 12	54 (54)	48 (48)	102 (51)	
*Gestational age (Weeks)*				
≤ 13	46 (46)	40 (40)	86 (43)	0.92
14-26	31 (31)	33 (33)	64 (32)	
≥ 27	23 (23)	27 (27)	50 (25)	

**Table-II:** The Mean and Standard Deviation of Psychiatric symptoms and Pregnancy Distress in Pregnant Women with and without Miscarriage History.

*Variables*	*Groups of study*			

	*with miscarriage history (N=100)*		*without miscarriage history (N=100)*	

*SCL-90-R*	*Mean*	*SD*	*Mean*	*SD*
Depression	12.33	7.87	7.69	7.43
Anexiety	8.89	6.41	5.65	4.69
Somatization	11.76	7.02	9.68	6.85
Obsessive – Compulsive	9.20	5.67	6.25	4.84
Interpersonal Sensitivity	6.23	4.97	4.77	4.78
Psychoticism	4.38	4.02	3.20	3.26
Paranoia ideation	4.62	3.52	3.12	3.11
Hostility	3.92	2.90	3.08	2.75
Phobic anxiety	3.73	3.26	3.15	3.06
GSI	0.77	0.43	0.55	0.41
*PDQ*				
Subscale 1	11.25	4.66	8.16	3.51
Subscale 2	3.12	2.15	2.52	2.23
Subscale 3	3.66	2.75	2.27	1.92
Total PDQ	18.03	8.60	12.95	6.69

*Ranges of scores:* Depression (0-52), Anexiety (0-40), Somatization (0-48), Obsessive–Compulsive(0-40), Interpersonal Sensitivity (0-36), Psychoticism (0-40), Paranoia ideation (0-28), Hostility (0-24), Phobic anxiety (0-28), subscale 1 (0-24), subscale 2 (0-12), subscale 3 (0-12), Total PDQ (0-48).

**Table-III:** Results of Multivariable analysis variance (MANOVA) tests of Psychiatric symptoms and Pregnancy distress in two groups

*Variables*	*Mean square*	*F*	*Significant*	*Partial Eta Squared*
*SCL-90-R*				
Depression	1076.48	18.35	<0.001	0.08
Anxiety	524.88	16.62	<0.001	0.077
Somatization	216.32	4.68	0.032	0.023
Obsessive – Compulsive	435.12	15.63	<0.001	0.073
Interpersonal Sensitivity	106.58	4.47	0.036	0.022
Psychoticism	69.62	5.17	0.024	0.025
Paranoia ideation	112.50	10.14	0.002	0.049
Hostility	35.28	4.40	0.037	0.022
Phobic anxiety	16.82	1.67	0.197	0.008
GSI	2.41	13.51	<0.001	0.064
*PDQ*				
Subscale 1	477.40	27.98	<0.001	0.124
Subscale 2	18	3.73	0.055	0.018
Subscale 3	96.60	17.07	<0.001	0.079
Total PDQ	1290.32	21.71	<0.001	0.099

## DISCUSION

Our data support the conclusion that pregnancy distress was higher in women with spontaneous abortion history than controls. A study confirmed that perinatal loss was associated with an increased likelihood of several mental disorders.^13^ In another study confirmed that patients who were initially more distressed continued to be disturb throughout the 1-year course.^4^ A study showed that women with a spontaneous abortion history reported significantly higher pregnancy-specific anxiety at first trimester than women with no history of spontaneous abortion.^11^

Pregnant women with spontaneous abortion history reported higher psychiatric symptoms and pregnancy distress than women without spontaneous abortion history. First, because spontaneous abortion may also put in doubt a woman’s sense of self-worthiness. Second, women who are pregnant after spontaneous abortion can have the feeling of lost control. Third, approximately 50% of women who have spontaneous abortion may conceive within a year following their loss. Studies have emphasized that the effects of spontaneous abortion on women’s anxiety levels during a subsequent pregnancy are of great interest.^14^-^16^ Hughes et al.^17^ found that the most recently bereaved women, and specifically those who had conceived within a year following a late pregnancy loss were at increased risk, in comparison to the control group. Another researcher found that at two-year’s post-spontaneous abortion, 26% of their participants were still experiencing clinically elevated levels of anxiety.^18^ As stress of women during pregnancy is associated with psychosocial factors,^19^ anxiety of the physical experience of spontaneous abortion can for some women have a significant cognitive and behavioral effect, resulting in avoidance and intrusive thoughts. A recent RCT reported that psychotherapy can reduce the complications of the pregnancy.^20^

### Limitations of the study

First, the cross-sectional nature of our study prevents any conclusion regarding causality. Second, this study did not assess psychiatric disorders in terms of clinical significance, but only in relation to specific pregnancy concerns that women are experiencing, via utilization of the SCL-90 and PDQ.

In summary, this study has suggested that pregnant women with less than a year after spontaneous abortion history are at risk of psychiatric symptoms (depression, anxiety, somatization, obsessive-compulsion, interpersonal sensitivity, psychoticism, paranoid, hostility, and Global Severity Index) and pregnancy distress (concerns about birth and the baby and concerns about emotions and relationships) more than controls. This study supports those interventions for planning the post spontaneous abortion psychological care for women, especially women who wanted to be pregnant during 12 month after spontaneous abortion.
